# Plastid Transformation: New Challenges in the Circular Economy Era

**DOI:** 10.3390/ijms232315254

**Published:** 2022-12-03

**Authors:** Rachele Tamburino, Loredana Marcolongo, Lorenza Sannino, Elena Ionata, Nunzia Scotti

**Affiliations:** 1 CNR-IBBR, Institute of Biosciences and BioResources, 80055 Naples, Italy; 2 CNR-IRET, Research Institute on Terrestrial Ecosystems, 80131 Naples, Italy

**Keywords:** plant biofactories, genetic transformation, chloroplast, tobacco, recombinant proteins, industrial enzymes

## Abstract

In a circular economy era the transition towards renewable and sustainable materials is very urgent. The development of bio-based solutions, that can ensure technological circularity in many priority areas (e.g., agriculture, biotechnology, ecology, green industry, etc.), is very strategic. The agricultural and fishing industry wastes represent important feedstocks that require the development of sustainable and environmentally-friendly industrial processes to produce and recover biofuels, chemicals and bioactive molecules. In this context, the replacement, in industrial processes, of chemicals with enzyme-based catalysts assures great benefits to humans and the environment. In this review, we describe the potentiality of the plastid transformation technology as a sustainable and cheap platform for the production of recombinant industrial enzymes, summarize the current knowledge on the technology, and display examples of cellulolytic enzymes already produced. Further, we illustrate several types of bacterial auxiliary and chitinases/chitin deacetylases enzymes with high biotechnological value that could be manufactured by plastid transformation.

## 1. Introduction

Climate change and overpopulation urge a conversion to a bio-based economic model founded on the development of more sustainable and environmentally-friendly procedures. The new 2020 agenda for a sustainable European economy (EC-Green Deal) includes the Circular Economy (CE) Action Plan, which aims to accelerate the transition towards the CE, with policies empowering consumers, and governing waste generation and sustainable products (WBCSD (2020) “Circular Economy Action Plan (CEAP) 2020 summary for business. Implications and next steps”). Indeed, bio-based solutions are expected to ensure technological circularity in priority areas, such as agriculture, biotechnology, ecology, green industry, and energy. 

In this context, biomasses and in particular plant biomasses are extremely significant in terms of renewable and sustainable production, and their function as feedstocks for food and animal feed, bio-based products, and burning for renewable energy [1]. Their inedible parts are mostly composed of cellulose, hemicellulose and lignin, whilst the edible portions contain starch, free sugars, proteins, vegetable oils and secondary metabolites of high value. The recovery of all these compounds requires the development of a sustainable biorefinery [1]. 

In a circular economy model, the replacements, in industrial processes, of chemicals with enzyme-based catalysts are expected to reduce the use and handling of toxic compounds, and benefit the environment, working staff and costs [2]. The available commercial enzymes, obtained from mesophilic microorganisms, are characterized by optimal activity in a narrow range of conditions and generally show a low stability in the conditions in which industrial processes usually take place. For this reason, there is a considerable demand for more stable, better performing and cheaper biocatalysts; thus, this research area is focusing on the development of improved enzymes through different strategies, such as genetic engineering of the proteins, use of thermophilic or hyperthermofilic enzymes, and biotechnological production of recombinant enzymes by different hosts (e.g., bacteria, yeast, plants, etc.) [3,4,5]. In the last decades, the plant-based platform for the production of recombinant proteins has had a huge boost due to low production costs, and the development of several strategies to overcome the main limitations of the system [6,7]. Among them, the production of recombinant enzymes by plastid transformation offers multiple attractions to biotechnologists [8,9,10], including the potential for a very high protein accumulation level [11,12].

Here, we review the current knowledge on plastid transformation technology, including examples of plastid-based recombinant cellulolytic enzymes, and—in a circular economy approach—the new challenges for this platform, especially in a model system such as tobacco, to produce recombinant enzymes with interesting applications in the industry.

## 2. Plastid Transformation Technology

Higher plant plastids (e.g., green chloroplasts in photosynthetically active cells, red/orange/yellow chromoplasts in fruits and flowers, colorless plastids in storage organs), originating from endosymbiotic events, possess their own genome (plastome or ptDNA) ranging in size from 120 to 160 kb. It is generally represented as a circular double-stranded DNA molecule with a tetrapartite genome organization characterized by a large single copy region (LSC) and a small single copy region (SSC), separated by two inverted repeat regions (IRs) [13,14]. During the gradual integration of the acquired endosymbionts into the host cell, the ptDNA underwent an impressive size reduction due to gene loss and gene transfer to the nuclear genome. In fact, compared to its cyanobacterial ancestors, it retains only a small portion of genes (110–120) which can be classified into three major groups: protein synthesis genes (e.g., rRNAs, RNA polymerase subunits, tRNAs, ribosomal proteins), photosynthesis-related genes, and other genes. Although plastids share several features with bacteria, the regulation of gene expression is rather complex and is mainly controlled at post-transcriptional level, in particular during the initiation phase of translation [9,13].

The small size of the plastome, the transgene containment conferred by maternal plastid inheritance in most crops, the precise transgene integration through a double homologous recombination event, and the extraordinary capacity to accumulate recombinant protein at high level, represent the most attractive features of this technology. Although many studies described successful production of foreign proteins in chloroplasts, the expected results were not achieved for all attempts. Many factors, such as the choice of expression elements (e.g., promoter, 5′- and 3′-untranslated region, UTR), the coding sequence, protein stability, localization, etc., can determine the efficiency of plastid transgene expression [15]. Most studies have been focused on the use of different regulatory sequences to optimize protein yield and stability [14,15,16], whilst less attention has been addressed to the protein localization in different sub-compartments of chloroplasts, considering the recombinant proteins produced so far mainly accumulated in the stroma. 

Chloroplasts have very complex structures: the envelope divides the organelles from the cytoplasm, it includes an intermembrane space between the outer and inner membranes and is the site of TOC-TIC translocation systems; the stroma, the major compartment of the chloroplast, harbors most of the plastid metabolic reactions, transcription, and translation processes; thylakoids are an interconnected network of membranes within the stroma and are the location of photosynthetic machinery; further, the last compartment contains either the thylakoid lumen involved in the folding/proteolysis of thylakoid proteins and protection against oxidative stress, or plastoglobules which are lipid droplets attached to thylakoid membranes acting in different aspects of lipid metabolism [17,18,19]. Few studies demonstrated that a precise intra-plastid targeting of some recombinant proteins, based on their own properties, can be pursued to improve both protein accumulation and functionality [17,18,19,20,21,22]. For example, Bally et al. [20] verified that both the stroma and the thylakoid lumen were competent in expressing a bacterial alkaline phosphatase enzyme. This enzyme’s activity was linked to the correct formation of two intramolecular disulfide bonds, but the targeting of the recombinant protein to the thylakoid lumen, mediated by its own secretion-type signal peptide, resulted in a larger accumulation and a more active enzyme. The thylakoid lumen was revealed as the right sub-compartment of chloroplast, also for the disulfide bond-containing aprotinin, although its yield was up to 0.5% of total soluble protein (TSP) [18]. Different results have been obtained when recombinant proteins were targeted to thylakoids. The chloroplast expression of the plastid terminal oxidase 1 from *Chlamydomonas reinhardtii*, a membrane protein involved in oxidation of plastoquinol pool, demonstrated the targeting of the recombinant protein to thylakoids in an enzymatically active form; although, the transplastomic plants showed a higher sensitivity than wild type plants to high-light [21]. On the contrary, in transplastomic plants expressing the bacterial outer surface protein A of *Borrellia burgdorferi* and the Pr55^gag^ polyprotein of human immunodeficiency virus (HIV-1), the thylakoids’ localization of recombinant proteins, due to their own sequences (i.e., signal peptide and highly basic region), produced severe mutant phenotypes [23,24]. Although the choice of targeting the recombinant proteins to a specific sub-compartment, based on their chemical-physical features, may improve their yield, stability and biological activity, an experimental validation is always needed. 

**Figure 1:**
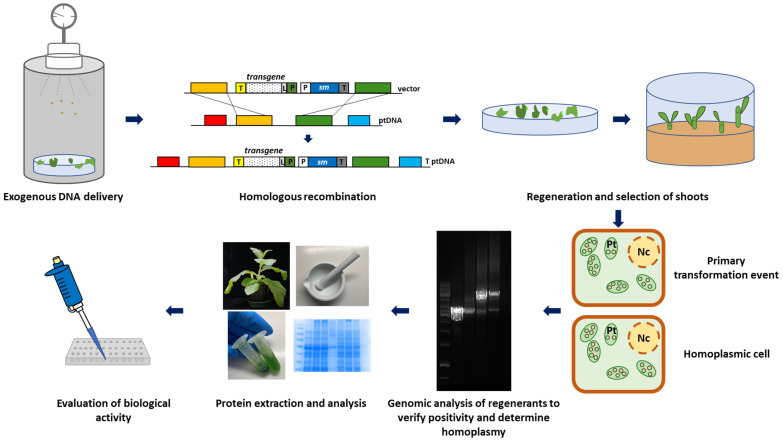
Schematic representation of chloroplast transformation in higher plants. Exogenous DNA delivered by biolistic method is integrated onto plastome following recombination events. Regeneration under selective pressure allows to determine homoplasmic lines that are further analyzed by molecular techniques.

From a biotechnological point of view, the methodology to transform the plastome is almost unchanged, notwithstanding some recent developments that have been published and will be described in the next paragraph. As summarized in Figure 1, gold particles are coated with exogenous DNA and bombarded in living cells and tissues by a gene gun. This methodology to introduce foreign DNA, known as the biolistic transformation method (the name is derived by fusion of biological and ballistic terms), is still the method of choice for plastid transformation. The integration of the transgene expression cassette into the ptDNA occurs through a double homologous recombination event. Since the primary transformation events involve only few copies of plastome (heteroplastic state), subsequent rounds of cell and organelle division (regeneration under selection pressure) are necessary to eliminate the wild type copy of the plastid genome, and thus achieve the homoplasmic state where all copies of ptDNA are transformed. To date, the most efficient selectable marker gene, conferring resistance to spectinomycin, is the *aadA*; its superiority is related to the specific action of spectinomycin as an inhibitor of plastid translation and low side effects compared to other cellular processes [10].

The subsequent steps of plastid transformation (Figure 1) are the molecular analyses (e.g., Southern blotting, PCR, etc.) of regenerants to select transformed (transplastomic) and homoplasmic plants. After the identification of homoplasmic plants, they will be evaluated for transgene expression using different assays (e.g., qRT-PCR, Western blotting, Elisa, spectrophotometry, etc.) to demonstrate the production level and the biological activity of the recombinant protein [13].

## 3. New Advances in Plastid Expression of Recombinant Proteins

**Figure 2:**
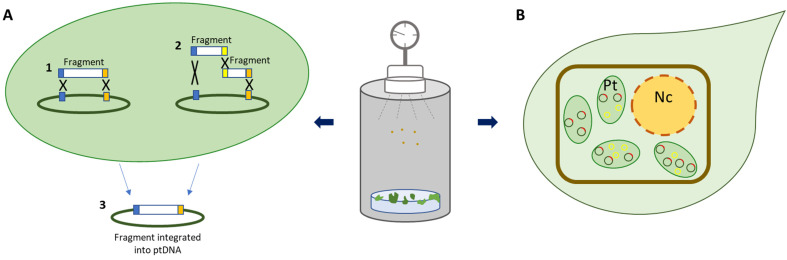
New approaches to express foreign proteins in plastids. (**A**) Fragmented DNA-mediated plastid transformation technology. Transgene may be directly delivered to chloroplast as a single (1) or two fragments (2) by biolistic method. In both cases, integration of transgene is mediated by two (or three) crossover events (3). Blue, yellow and orange boxes are homologous sequences. X indicates homologous recombination events. (**B**) Minichromosome-based approach for chloroplast transformation. Plastid transformation vectors based on geminivirus DNA amplification system were developed and delivered to chloroplast through biolistic method. Minichromosomes are reported as yellow circles; plastid DNA containing *rep* transgene as green-red circles. Pt = plastids, Nc = nucleus.

Over the last few years, several attempts have been made to simplify and/or shorten the chloroplast transformation procedure. As already stated, traditional plastid transformation requires the construction of a vector that could be a time-consuming step since no standard vectors are available. To overcome such an issue, Ren, et al. [25] described a simple technology for plastid transformation, by using linear fragmented DNA having homologous sequences (HSs) at their ends. Multiple linear DNA fragments with HSs of different sizes (i.e., 500, 200 and 50 bp) at their ends were generated from plasmid pYY12, containing the *gfp* (i.e., green fluorescent protein) expression cassette [26], and delivered through the biolistic method as a single fragment or in a combination of two fragments (Figure 2A). The integration onto the plastome is mediated by two or three crossover events. The length of HSs and linear DNA structure had a positive effect on the efficiency of transformation, as compared with that of the entire plasmid pYY12. All transplastomic plants reached the homoplasmic state after two rounds of regeneration on a selective medium and showed a phenotype similar to wild type plants when grown under greenhouse conditions. Reciprocal crosses between transplastomic and wild type plants definitively demonstrated the transformation of ptDNA, excluding nuclear transformation. Ren, et al. [25] successfully applied their approach to express a bacterial phage lytic enzyme (plyGBS) in tobacco chloroplasts obtaining a protein yield up to 50% TSP, but transplastomic plants showed a mutant phenotype likely due to the massive protein accumulation reached. Similar phenotypic alteration was observed when the same recombinant protein was produced in tobacco chloroplasts by using the classical plastid expression vector [11]. In this work, the authors demonstrated that the observed mutant phenotype was due to the impairment in the production of endogenous chloroplast proteins, by exhaustion of plastid translation machinery originating from massive production of plyGBS (up to 70% TSP) [11]. Furthermore, the linear fragmented DNA-based technology was also used to express three Arabidopsis photosynthesis-related genes as polycistron, but gene expression was verified only at transcript level. Notwithstanding, the procedure described offers a valuable starting point to the development of a vector-free plastid transformation; further studies are required to verify whether more than two fragments may be used for plastid transformation. In addition, the need to verify the DNA fragment(s) through sequencing appears to be a weakness of this method. 

The achievement of homoplasmy represents, for some species, a time-consuming and difficult step. To overcome such limitations, Jakubiec, et al. [27] have developed a plastidial transformation vector based on geminivirus replication (i.e., minichromosome), containing viral origin of replication (VOR) sequences from beet curly top geminivirus (BCTV). They successfully used this vector to transform a wild type and a transplastomic tobacco plant expressing the replication initiator protein (Rep) (Figure 2B). Although the stable and efficient amplifications of foreign DNA (i.e., *gfp*) were demonstrated in both plants, the best results in terms of phenotype (i.e., mild yellowing) and recombinant protein yields (four-fold higher compared to results with the classical transformation method) were obtained with transplastomic Rep plants. Therefore, it is conceivable that to obtain encouraging results, this technology needs a transplastomic plant expressing Rep protein as the plant host.

Very recently, a new strategy has been developed based on a fusion peptide KH-AtOEP34 containing the polycationic DNA-binding (KH)_9_ sequence, and the plastid-localizing peptide of outer envelope membrane proteins (OEP34/TOC34), to deliver exogenous DNA to the chloroplast [28]. The novel approach was applied to three species: the model plant tobacco, and two crops (rice and kenaf) for which no efficient plastid transformation protocol has been previously described. The integration of exogenous DNA onto ptDNA mediated by homologous recombination events was verified, but with very low transformation efficiency and no homoplasmy was obtained even with tobacco. Further, the authors could not exclude the integration of exogenous DNA onto nuclear DNA. Strengths and weaknesses of the described methods are summarized in Table 1.

## 4. Plastid-Based Enzymes for the Biorefinery Industry

To date, several bacterial and fungal cellulolytic enzymes have been produced using tobacco plastid transformation technology (Table 2). They are mainly glycoside hydrolase enzymes able to process cellulose and hemicellulose backbones from the ends of polymer chains (e.g., exoglucanases), acting on internal glycosidic bonds (e.g., endo-glucanases and xylanases), and hydrolyzing cellobiose releasing D-glucose (i.e., β-glucosidases). In addition, catalytic activities able to act on lignin and more complex substrates are also reported (e.g., swollenin, cutinase, β-mannase, pectate lyases, manganese peroxidase). Among diverse activities required to the complete depolymerization of lignocellulosic biomass, β-glucosidase and xylanase, from both bacterial and fungal sources, are the most reported to be expressed in transplastomic tobacco. 

As there are concerns about xylanases, several regulatory elements were used to express the corresponding transgenes in chloroplasts. Particularly, Leelavathi, et al. [29] and Verma, et al. [30] used the 5′- and 3′-untranslated region (UTR) of the plastidial *psbA* gene to express a xylanase from *Bacillus subtilis* and *Trichoderma reesei*, respectively, with a protein yield up to 6% TSP for xylanase from *B. subtilis*. Regulatory regions containing both UTR and the first 10–14 codons of coding sequences (downstream box, DB) were used to reach high protein accumulation by improving translation efficiency [16] for the expression of thermophilic [31] or hyperthermophilic [12] xylanases. The use of 5′-UTR and DB sequences of the plastid *atpB* gene produced a yield up to 36% TSP for the thermophilic xylanase from *Alicyclobacillus acidocaldarius*, which is the best accumulation level reported for this enzyme to date; whilst the 5′-UTR of *T7g10* and the *gfp* DB resulted in 15% TSP accumulation for GH10 xylanase from *Thermotoga maritima* [31]. In a comparative study, Kolotilin, et al. [32] evaluated a set of expression cassettes differing in regulatory elements (5′-UTR and DB) to produce the bacterial xylanase XynA from *Clostridium cellulovorans*. The most productive cassette containing the *T7g10* regulatory sequences yielded the highest accumulation (up to 0.5% TSP) of XynA and produced a normal plant phenotype. Therefore, it was further used to express two fungal xylanases, Xyn10A and Xyn11B from *Aspergillus niger*, giving contrasting results in terms of yield for the two recombinant proteins (i.e., 0.2 and 6% TSP, respectively). The removal of such regulatory sequences allowed for an increase in the accumulation of Xyn10A of up to 2% TSP, while Xyn11B levels decreased to 2.5% TSP. In all cases, transplastomic plants expressing xylanases showed normal phenotypes, resulting in them being indistinguishable from the wild type, independently of the expression level obtained. 

The plastid-based xylanases retained their main features, even showing improved activity, and were able to depolymerize several complex matrices (e.g., oat spelt, poplar, pine wood, orange peel, *Arundo donax*) [12,29,30,31] in combination with other commercial enzymes. As an example, the xylanase from *T. reesei* showed a five-fold increased activity as compared to the *E. coli*-expressed form [30], whereas the xylanase from *A. acidocaldarius* was more thermophilic than the counterpart expressed in *E. coli* [12], thus more appealing for industrial purposes. Importantly, for several plastid-based xylanases, it has been demonstrated that they have the ability to retain enzymatic activity even when the leaves are dried, thus facilitating transport and storage steps [29,31]. 

**Table 2:** Expression of lignocellulolytic enzymes in chloroplasts of higher plants.

Heterologous Protein	Gene	Source	Expression Level	Phenotype of Transplastomic Plants	Physico-Chemical Characterization	References
acetyl xylan esterase	*axe1*	*Trichoderma reesei*	color change observed	wild type	-	[30]
β-glucosidase	*bgl1*	*Trichoderma reesei*	14 U/mg CTSP ^a^	wild type	50 °C, pH 5.2 ^b^	[30]
β-glucosidase	*bglC1*	*Thermobifida fusca*	5–40% TSP ^c^	mutant	T_opt_ 45 °C, pH_opt_ 7.0	[33]
β-glucosidase	*bglC*	*Thermobifida fusca*	up to 12% TSP	nd ^d^	50 °C ^b^	[34]
β-glucosidase	*celB*	*Pyrococcus furiosus*	up to 75% TSP	wild type	T_opt_ 105 °C, pH 4.0–5.0	[12]
β-glucosidase	*bgl1*	*Aspergillus niger*	20 mg/g TSP	wild type	T_opt_ 40 °C, pH 5.0	[35]
β-mannase	*man1*	*Trichoderma reesei*	25 U/g FW	mutant (pale green)	40 °C < T_opt_ < 70 °C, 3.0 < pH_opt_ < 7.0	[36]
cellulases	*celA*	*Thermotoga neapolitana*	23 mg/g TSP	wild type	T_opt_ 65 °C, pH 5.0	[35]
cellulases	*celB*	*Thermotoga neapolitana*	21 mg/g TSP	wild type	T_opt_ 65 °C, pH 5.0	[35]
cutinase	*cut*	*Fusarium solani*	15 U/mg CTSP	wild type	30 °C, pH 8.0 ^b^	[30]
cutinase	cut	*Trichoderma reesei*	nd	mutant	nd	[37]
endo-1,4-β-glucanase	*cel6A*	*Thermobifida fusca*	up to 2% TSP	nd ^b^	50 °C ^b^	[38]
endoglucanase	*cel6A*	*Thermobifida fusca*	up to 10% TSP	wild type	nd	[39]
endo-β-glucanase E1 catalytic domain (E1cd)	*e1cd*	*Acidothermus cellulolyticus*	12% TSP	mutant	55 °C ^b^	[40]
endoglucanase	*celD*	*Clostridium thermocellum*	up to 4930 U/g FW	wild type	T_opt_ 60 °C, pH_opt_ 6.0	[30]
endoglucanase	*egI*	*Trichoderma reesei*	339 U/mg CTSP	wild type	50 °C, pH 5.2 ^b^	[30]
endoglucanase	*cel9A*	*Thermobifida fusca*	40% TSP	mutant	nd	[33]
endoglucanase	*egph*	*Pyrococcus horikoshii*	25% TSP	mutant (pale green)	85 °C, pH 5.5 ^b^	[41]
endoglucanase	*cel7*, *endoV*, *celK1*	*Chaetomium globosum*, *Paenibacillus sp.*	8–10% TPC ^e^	nd	60 °C ^b^	[42,43]
endoglucanase	*endo*	*Sulfolobus solfataricus*	2% TSP	mutant (severe retarded growth)	nd	[12]
exo-cellobiohydrolase	*cel6B*	*Thermobifida fusca*	3% TSP	nd ^b^	50 °C ^b^	[38]
exo-cellobiohydrolase	*cel3*	*Phanerochaete chrysosporium*	0.4 U/mg protein	nd	nd	[42,43]
exoglucanase	*celO*	*Clostridium thermocellum*	442 U/mg CTSP	wild type	60 °C, pH 5.2 ^b^	[30]
exoglucanase	*cel6B*	*Thermobifida fusca*	5% TSP	mutant	nd	[33]
exoglucanase	*cel6*	*Chaetomium globosum*	0.4 U/mg protein	nd	nd	[42,43]
lipase	*lipY*	*Mycobacterium tuberculosis*	nd	wild type	-	[30]
manganese peroxidase	*mnP-2*	*Phanerochaete chrysosporium*	nd	normal (high growth rate)	65 °C, pH 6.0	[44]
pectate lyases	*pelA*, *pelB*, *pelD*	*Fusarium solani*	up to 32 U/g FW	wild type	T_opt_ 40 °C, 6.0 > pH_opt_ > 8.0	[30]
pectinase	*pelA*	*Streptomyces thermocarboxydus*	nd	wild type	60 °C, pH 7.0	[44]
swollenin	*swo1*	*Trichoderma reesei*	swelling observed	wild type	-	[30]
swollenin	swo1	*Trichoderma reesei*	nd	mutant	nd	[37]
xylanase	*xynA*	*Bacillus subtilis* strain NG-27	6% TSP	wild type	T_opt_ 70 °C pH_opt_ 8.4	[29]
xylanase	*xyn2*	*Trichoderma reesei*	421 U/mg CTSP	wild type	50 °C, pH 5.2 ^cb^	[30]
xylanase	*xyl10B*	*Thermotoga maritima*	up to 15% TSP	wild type	termostable at 85 °C for 30 min	[31]
xylanase	*xynA*	*Clostridium cellulovorans*	0.5% TSP	wild type	40 °C ^b^	[32]
xylanase	*xyn10A*	*Aspergillus niger*	up to 3% TSP	wild type	40 °C ^b^	[32]
xylanase	*xyn11B*	*Aspergillus niger*	up to 6% TSP	wild type	40 °C ^b^	[32]
xylanase	*xyn*	*Alicyclobacillus acidocaldarius*	36% TSP	wild type	T_opt_ 80 °C pH 6.0	[12]
xyloglucanase	*xeg74*	*Thermobifida fusca*	5–40% TSP	mutant (pigment deficiency, retarded growth)	nd	[33]

^a^ CTSP crude total soluble protein. ^b^ assay conditions. Temperature and pH optima were not determined. ^c^ TSP total soluble proteins. ^d^ not determined. ^e^ TPC total protein content.

The expression levels of β-glucosidases produced in transplastomic plants ranged from 1.6 to 75% TSP [12,30,33,34,35]. Petersen and Bock [33] expressed a β-glucosidase (*bglC*) from *Thermobifida fusca* using the T7g10 5′-UTR, and obtained high protein yield and plant mutant phenotype. Since the high yield obtained did not explain, alone, the mutant phenotype, the authors speculated that it was due to carbohydrate-binding activity of the recombinant protein; this might interfere with the carbohydrate metabolism of the chloroplast and suggest that a lower accumulation might be less unfavorable to plant growth and phenotype. 

Concomitantly, Gray et al. [34] described the development of three expression cassettes, differing from those of Petersen and Bock [33], for the presence of the DB sequences originating from the *tetC*, *nptII* and *gfp* genes fused with *bglC* ORF. Transplastomic plants with NPTII-BglC and TetC-BglC proteins accumulated up to 12 and 2.6% TSP, respectively; no protein accumulation was detected in GFP-BglC plants. The normal phenotype observed for these transplastomic plants supported the hypothesis formulated by Petersen and Bock [33] that a lower accumulation of BGL protein did not interfere with plant phenotype. 

Verma et al. [30] and Jin et al. [45] reported the expression of a β-glucosidase from *T. reesei* under the control of the 5′- and 3′-UTRs of the *psbA* gene. The enzyme revealed a seven-fold increase in its activity, as compared to the *E. coli*-expressed form [30], and beneficial effects on plant phenotype, including increased height, biomass, leaf area, a higher production of phytohormones (e.g., giberellin, zeatin), and trichome density as compared to wild type [45]. Similarly, the expression of β-glucosidase from *Aspergillus niger* was regulated by a 5′-UTR from the *rrn16* gene and a 3′-UTR from the *rbcL* gene, and produced a 1.4-fold improved enzymatic activity [35] compared to wild type plants, and twice that reported by Verma, et al. [30]. In the same year, Castiglia et al. [12] produced a β-glucosidase from the hyperthermophilic *Pyrococcus furiosus* using two expression cassettes differing in 5′-UTR and DB regulatory sequences from plastidial *atpB* and *rbcL* genes (pDC21 and pDC23), respectively. Very high accumulation levels were reached with both constructs (≥75 and 60% TSP, respectively) and with different plant phenotypes. The DC21 line showed a mutant phenotype, with retarded growth due to an exhaustion of plastid translation machinery originating from massive protein production; this was confirmed by the normal phenotype observed with the transplastomic line (DC23) accumulating β-glucosidase at lower level (≥60% TSP). As for xylanase, plastid-expressed β-glucosidases also maintained their native properties. Particularly, *P. furiosus* β-glucosidase showed a pronounced thermophilicity, retaining 66% of activity at 120 °C, whereas the native form rapidly lost its activity above 105 °C [12]. In addition, plastid-based β-glucosidases boosted depolymerization of complex biomasses (e.g., orange peel, pine wood, filter paper, tobacco) in association with commercial cellulases [30,34].

Overall, several features make the recombinant enzymes more appealing for industrial purposes. Among them, stability and activity at industrial processing conditions, meaning high temperatures, and acidic or basic pHs, suggests that enzymes from extremophiles are more suitable for such purposes. As an example, the high production levels, and the intrinsic properties of plastid-based β-glucosidase and xylanase from hyperthermophilic *P. furiosus* and thermophilic *A. acidocaldarius*, allowed for a streamlined and cost-effective enrichment strategy that satisfies industrial requirements.

## 5. New Challenges for Biocatalysts and the Circular Economy

### 5.1. Enzymes for Lignocellulosic Waste Biorefinery

**Figure 3:**
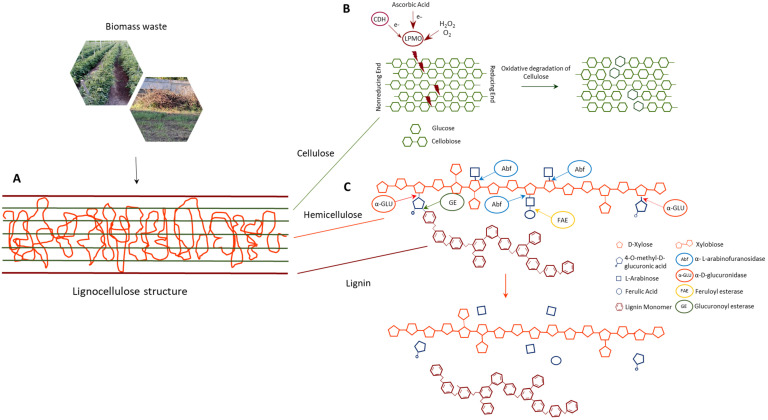
(**A**) Schematic structure of lignocellulosic biomass. (**B**) Action of LMPOs on crystalline cellulose chain by oxidative degradation. (**C**) Action of accessory enzymes on hemicellulose, with the cleavage of glycosidic and ester bonds between xylan and its side chains, and of ester bonds between hemicellulose and lignin.

In the circular economy perspective, lignocellulose exploitation follows biorefinery concepts that involve the utilization of all the lignocellulosic biomass components. Only bio-based processes, realized by the application of enzymes instead of the classical methods, allow the lignocellulosic complexes to be disintegrated; this leads to polysaccharide bioconversion into simple sugars for the production of biofuels and chemicals, and the extraction of bioactive molecules with high biotechnological value [46]. The fundamental task of (hemi)cellulolytic enzymes, active on linear polysaccharides chains, is greatly improved by biocatalysts able to break down the chemical linkages (Figure 3) which strictly interconnect the different plant cell wall polymers. These enzymes, commonly named auxiliary enzymes, have a relevant role in substituting traditional chemical pretreatments, aiding in fermentable sugar recovery and allowing the extraction of powerful bioactive compounds, mainly of polyphenolic nature, that crosslink plant cell wall components [47]. Their biotechnological relevance makes their expression, through the transplastomic platform, of particular interest in a circular economy perspective. The possibility to obtain high protein yields, by plants as biofactories that utilize atmospheric CO_2_ through photosynthesis, offers a cost-effective valuable alternative to the traditional reactor-based system with low environmental impact. Considering the cyanobacterial origin of chloroplasts, it is not surprising that enzymes from bacterial microorganisms fit well with plastid translation machinery. Therefore, reported in the following sections are the most promising auxiliary enzymes, never expressed in plants from bacterial and archaeal microorganisms, that could represent the new challenges of plastid transformation. Furthermore, the selected enzyme classes were expressed in bacterial hosts with high yields, without revealing particular toxicity profiles or problems in folding.

#### 5.1.1. Lytic Polysaccharide Monooxygenases

Copper-dependent lytic polysaccharide monooxygenases (LPMOs) are known as Auxiliary Activity (AA) families AA9-11 and AA13-16 in the Carbohydrate-Active enZymes (CAZy) database [48], on account of their activity on polysaccharides. These boosting enzymes preferentially degrade polysaccharides characterized by high crystallinity, such as cellulose and chitin, but also hemicellulose, pectin, and starch [49]. LPMOs have recently assumed the role of key enzymes in the best-performing cellulolytic mixtures, and there are numerous studies aimed at identifying and optimizing these challenging biocatalysts.

LPMOs are distributed in most kingdoms of living organisms and are found in bacteria, archaea, viruses, and eukaryotes, including fungi, plants, and animals. Numerous studies have investigated fungal LPMOs, but bacterial LPMOs are still under exploration [48]. The first LPMO identified was the mono-copper oxidase from *Serratia marcescens*. The bacterium was capable to produce a chitin-active LPMO, formerly identified as Chitin Binding Protein (CBP) 21 [50], responsible for efficient chitin degradation [51]. The discovery of CBP21 was the key to identifying the novel family known as LPMOs.

Streptomycetes, soil microorganisms living on decaying lignocellulosic materials, are recognized as important LPMOs producers [52]. Among them, *S. coelicolor* A3 is the best-studied representative of this group, with seven LPMOs encoded by its genome [53] and heterologously expressed in *E. coli*.

Auxiliary activity 10A (TfAA10A) from *T. fusca*, a thermophilic soil bacterium with a good growth at 50 °C on cellulose, was cloned in *E. coli* [54]. Recently, this enzyme was also overexpressed in the cyanobacterium *Synechococcus elongatus* [55]. The first study on the expression and identification of a LPMO (NaLPMO10A) from *Natrialbaceae archaeon* [56], a halophilic alkalithermophilic archaeon, resulted in high interest. NaLPMO10A showed thermostability and pH stability under alkaline conditions (pH 9.0), and metal ions (Na^+^, K^+^, Ca^2+^ and Mg^2+^) clearly enhanced the efficiency of chitin degradation.

The LPMOs ability to improve the hydrolysis efficiency of classical biocatalysts, on several lignocellulosic materials, implies an important possibility of reducing the enzymatic loads useful for the process of converting biomass into biofuel [49].

#### 5.1.2. Arabinofuranosidases

Arabinofuranosidases (Abfs) remove L-arabinofuranosyl residues present on the side chains of xylan, with a high variation in terms of substrate preference and hydrolyzed linkages. Abfs are high value biotechnological tools in several industrial processes, including the synthesis of oligosaccharides [57], the hydrolysis of lignocellulosic biomass [58] and in the pulp and paper industry as an alternative to chemical chlorination [59]. Moreover, α-L-arabinofuranosidases are involved in the production of drug compounds [60] and improvement of some food properties, such as wine flavor and juice clarification [61]. In particular, Abfs specifically catalyze the hydrolysis of α-1,2, α-1,3, α-1,5 glycosidic bonds involving L-arabinofuranosyl residues [62]. In the CAZy database, the Abfs belong to the Glycosyl Hydrolase (GH) families 2, 3, 10, 43, 51, 54, and 62 [63]. Various arabinofuranosidases have been identified in several plants, fungi and bacteria, providing useful information for enzymatic characterization. In this context, the thermotolerant enzymes show a better stability and industrial applicability, and the thermophilic bacteria are major producers of the aforementioned biocatalysts [3].

Streptomycetes have been found to be the most abundant hemicellulases producer among actinomycetes. *Streptomyces thermoviolaceus* is able to produce a GH62 α-L-arabinofuranosidase (SthAbf62A) capable of withstanding high temperature (60 °C) and producing reducing sugars from polymeric arabinoxylan [64]. Recently, an Abf purified from *Streptomyces lividus* revealed interesting characteristics, resulting stable over a broad pH range of 3.0–11.0, and temperature of 30–80 °C, with an optimum temperature of 60 °C and pH 9.0 [65].

Two GH51 Abfs, Abf1Geo12 and Abf2Geo12 from the thermophilic bacterium *Geobacillus stearothermophilus* 12, showed relevant characteristics. Indeed, Abf1Geo12 and Abf2Geo12 resulted active in a broad range of pH (5.0–9.0) and temperatures of 50–85 °C and of 40–80 °C, respectively [66]. Comparable characteristics were found in *Geobacillus vulcani* GS90 α-L-arabinofuranosidase (GvAbf) that exhibits an optimum activity at 70 °C and pH 5.0 when expressed in *E. coli* [67]. Moreover, the studies conducted in fruit juice of apple, grape, peach and orange pulps, revealed that GvAbf and xylanase, working in synergy, implemented the reducing sugar level useful in fruit juice enrichment processes [66].

A performant hyperthermostable Abfs (Tt-Afs) gene from *Thermotoga thermarum* DSM5069, expressed in *E. coli*, showed an optimal activity at pH 5.0 and temperature of 95 °C [68].

The GH43 family has provoked particular interest and has many multifunctional xylanolytic enzymes, including bifunctional β-xylosidase/α-L-arabinofuranosidases. The bifunctional β-xylosidase/α-L-arabinosidase from the Archea *Sulfolobus solfataricus* P2 was expressed in *E. coli* [69]. The β-xylosidase retained 100% activity at 80 and 90 °C, while 100% activity of the Abfs was observed at 80 °C. A bifunctional GH43 α-L-arabinofuranosidase/β-xylosidase (CAX43) from *Caldicellulosiruptor saccharolyticus* DSM8903 [70] displayed maximum activity at pH 6.0 and 70 °C. These enzymes are important for boosting xylo-oligosaccharide hydrolysis efficiency in biomass conversion, reducing production costs [71]. Additionally, the multifunctional enzymes are important, such as GH43 α-L-arabinofuranosidase/endoxylanase/β-D-xylosidase. In particular, *Paenibacillus curdlanolyticus* B-6 produced PcAxy43B, is capable of releasing arabinose, xylose, and XOSs from the arabinoxylan [63].

#### 5.1.3. α-Glucuronidases

Glucuronic acid is a component of the structural polysaccharides that decorate the hetero-xylan polymer. D-glucuronic acid or its methyl derivative, 4-O-methyl-D-glucuronic acid bound to the C-2 positions of the D-xylose residues, are present in glucuronoxylans; when L-arabinofuranosyl moieties are also linked to xylan, the polymer takes the name of arabinoglucuronoxylans [72]. Glucuronic acid residues can form ester linkages to the hydroxyl groups of lignin, providing cross-links between the cell wall, and the lignin [72]. The strong bond with glucuronic acid can be hydrolyzed by α-glucuronidases, belonging to the families GH4, GH67 and GH115 of the CAZy database [73].

Despite the importance in nature of the glucuronic acid substituents, there are very few known α-glucuronidase encoding genes (<40), most of which are from fungi and bacteria [74]; while no archaeal α-glucoronidases have been identified so far.

Among the thermophilic bacteria, in *Clostridium stercorarium* and *Thermoanaerobacterium saccharoliticum* intracellular α-glucuronidase activities were found [75]. They showed a similar optimum pH (pH 5.5–6.5), but the *C. stercorarium* enzyme was more stable at 60 °C with a half-life of 14 h, almost 6-fold higher than *T. saccharoliticum* enzyme.

Two different strains of *Geobacillus stearothermophilus*, T6 and 236, produced α-glucuronidases belonging to the GH67 family, with optimal activity between 40 and 65 °C and pH 5.5–6.5 [76,77]. The biocatalyst of *G. stearothermophilus* 236 acts mainly on small substituted xylo-oligomers and is able to increase the yield of xylose from xylan, in combination with endoxylanase and β-xylosidase [76].

The hyperthermophilic bacterium *Thermotoga maritima* possess a gene encoding a thermoactive GH67 α-glucuronidase. This enzyme showed highest activity at 85 °C and pH 6.3, and produced xylobiose and 4-O-methylglucuronic acid [78]. The GH4 α-glucuronidase from *T. maritima*, active at 80 °C and pH 7.0, was also reported [79].

Glucuronoxylans represent a bottleneck in hemicellulose degradation. The use of α-glucuronidase, in synergy with other hemicellulolytic enzymes, facilitates the recovery of xylan. This process is also a crucial goal for biorefineries that rely on enzymatic degradation and the use of xylan in the production of biomaterials [80].

#### 5.1.4. Feruloyl Esterases

Ferulic acid (FA) and other hydroxycynnamic acids (HCAs), which are esterified to the arabinose units of arabino-xylan and to arabinan and galactan, establish numerous inter-molecular connections among the polysaccharide chains and lignin. Feruloyl esterases (FAEs), cleaving most of these ester bonds, liberate FA and/or HCAs facilitating the release of fermentable sugars by glycosyl hydrolases [81,82]. For its recognized powerful antioxidant and antimicrobial activities, extensive studies address ferulic acid applications in several sectors such as food, medicine, pharma, water treatment, and cosmetic [83]. Further, FAEs-mediated transesterification reactions, that allow for improvement of FA solubility in water and lipophilic media by its transformation in feruloylated compounds, are of great biotechnological interest [84]. FAEs are classified as carbohydrate esterases and belong to a subclass of family CE1 of the CAZy database [72]. Although, still today, Fungi are considered as the most important producers of these enzymes, studies on FAEs from bacterial sources have already started since the nineties, discovering biocatalysts with relevant biotechnological properties. Important FAE producers are represented by Lactobacilli. Two FAEs from *L. plantarum* strains are considered good candidates for biotechnological applications due to their broad substrate specificity, one of them being able to work also on model substrates for tannases [85]. Six FAEs were described from *L. fermentum* JN248, some of which showed a good level of stability at high temperatures and pH, making these biocatalysts suitable for application in pulp and paper treatment. Moreover, the recovery of up to 70% of alkali extractable ferulic acids from de-starched wheat bran were obtained with the utilization of these FAEs in combination with a xylanase [86].

*L. helveticus* KCCM 11223 and *L. acidofilus* F46 also produce thermostable cinnamoyl esterases. The enzymes, optimally active at elevated temperatures (65 and 50 °C, respectively), were able to hydrolyze chlorogenic acid, releasing caffeic acid, with powerful antioxidant activity [87,88].

*Geobacillus* and *Thermobacillus* genera have also been studied as producers of thermophilic FAEs. The feruloyl esterase from *G. thermoglucosidasius* DSM 2542 (GthFAE), preferentially worked at pH 8.5 and 50–60 °C showing elevated stability at these conditions; thus, its main suggested application was in the pulp and paper sector [89]. Similarly, Tx-est1, the feruloyl esterase from *Thermobacillus xylanilyticus*, worked optimally at 65 °C and pH 8.5, retained 80% of its maximal activity at 80 °C and was stable at 50 °C for 24 h [90]. Thus, it was applied for the recovery of para-coumaric and diferulic acids from non-delignified wheat bran and straw, also in association with the xylanase from *T. xylanolyticus*, giving encouraging yields [90].

Feruloyl esterases also occur as multi-domain enzymes. High efficiencies in recalcitrant biomass degradation were verified for the two feruloyl esterases from *Bacteriodetes ovatus* and *Flavobacterium johnsoniae*, that present both acetyl esterase and FAE domains [91]. The other two bifunctional, multimodular FAEs were studied from *Clostridium thermocellum* that contained two xylanases’ domains (Xyn 10A and Xyn10B). The feruloyl esterase with a Xyn10A module recombinantly expressed in *E. coli,* revealed biotechnologically relevant features, being optimally active between pH 4.0 and 7.0 at 50–60 °C, and stable at 70 °C for 6 h [92,93].

#### 5.1.5. Glucuronoyl Esterases

Glucuronoylesterases (GEs) are carbohydrate esterases that cleave ester bonds between 4-O-methyl-D-glucuronic acid (MeGlcA) residues of glucuronoxylan and lignin. These biocatalysts, cleaving the covalent bonds between polysaccharides and lignin, are the main contributors to plant cell wall recalcitrance against the enzymatic attack [94]. They reduce the costs for polysaccharide bioconversion into simple sugars, and support the routes of lignin exploitation [95]. Moreover, GEs actions in synergy with (hemi)cellulolytic hydrolases [96,97] lead to the recovery of glucuronidated xylo-oligosaccharides, known for their antibacterial and anti-inflammatory action. For their transesterification activity, GEs are also recognized as synthetic tools for the production of alkyl-branched glucuronic acid esters, and non-ionic surfactant with interesting properties for several applications [98].

GEs were assigned to the novel CAZy family CE15 [99] and, although the highest number of GEs has been studied from saprophitic Fungi, bacterial glucuronoyl esterases began to be investigated, revealing a broader substrate specificity than their fungal counterparts.

A wide substrate spectrum was revealed for MZ0003, a single-domain bacterial GE with optimal activity in the presence of 1M NaCl, which was identified from a metagenome library of arctic marine sediments [100]. MZ0003, overexpressed in *E. coli*, worked on model substrates for glucuronoyl and acetyl esterase activities. MZ0003 could find applications for the recovery of bioactive compounds from marine carbohydrate polymers. The GEs ability to work on a wide spectrum of substrates was often ascribed to the co-presence of multiple domains. CesA, a bimodular acetyl xylan esterase-glucuronoyl esterase, studied by Aurilia, et al. [101] as component of the cellulosome from *Ruminococcus flavefaciens*, revealed a C terminal domain whose GE function was subsequently demonstrated by Biely et al. [102] after its expression in *E. coli*. A multiple domain glucuronoyl esterase (CkXyn10C-GE15A), containing a module with xylanase activity and others with carbohydrate binding function, was studied from the hyperthermophilic bacterium *Caldicellulosiruptor kristjansonii* [103]. CkXyn10C-GE15 is the most thermostable GE studied so far, with an optimal temperature around 72 °C.

Several bacteria were able to produce multiple glucuronoyl esterases with complementary specificities. Three genes encoding for GEs were identified in the genome of the bacterium *Teredinibacter turnerae*, a symbiont of marine wood-boring bivalve ship-worms [104]. Only one of them was expressed in a soluble form in *E. coli*, worked optimally at pH 8.5, and was involved in the breakdown of lignin carbohydrate complexes of marine lignocellulosic biomass. Three bacteria, namely *Opitutus terrae*, *Spirosoma linguale*, and *Solibacter usitatus*, had genomes containing, respectively, four, three, and three ORFs encoding putative GEs [94], with higher activity than their fungal counterparts.

### 5.2. Other Enzymes for Waste Biorefinery

In advancing the circular economy, valorization of waste from the fishing industry, an untapped resource, which is cause of serious environmental concern, can be turned into a valuable opportunity. Processed seafood waste is mainly constituted by chitin (20–58% of dry weight), the most abundant biopolymer of oceans biomass [105], which represents the principal component of the exoskeleton of arthropods (crustaceans), and the endoskeleton of mollusks. In the biorefinery of chitin, a crystalline polysaccharide consisting of linear chains of β-1,4 linked N-acetyl glucosamine units, the fundamental role is played by chitinases and chitin deacetylases—an alternative to the traditional chemical procedure. In the following sections, several studies on the most biotechnologically relevant chitinases and chitin deacetylases are summarized.

#### 5.2.1. Chitinases

Chitinases are glycosyl hydrolases included in families GH18, 19, and 20 of the CAZy database [106]. Chitinolytic enzymes are able to hydrolyze β-1,4-glucosidic linkages that connect N-acetyl glucosamine units of chitin chains. The biotechnological relevance of chitinases is widely recognized, mainly due to the beneficial properties of acetyl-glucosamine and chitooligosaccharides produced by their activity. These characteristics are at the basis of the numerous applications in medicine, food industry, biotechnology, agriculture, waste management, and crop protection sectors [107].

Several organisms express chitinases for different physiological functions, including fungi, bacteria, archaea, viruses, plants, insects, and crustaceans.

One of the first identified species involved in the degradation of chitin was *S. marcescens*. Able to produce [108] five different GH18 chitinolytic enzymes with maximum activity at 32 °C and pH of 8.0, *S. marcescens* is considered a model bacterium for the study of chitinolytic activities [109].

A thermophilic chitinase was produced by *Paenibacillus* sp. TKU052. The enzyme exhibited high catalytic activity at elevated temperature and acidic pH conditions (70 °C, pH 4.0–5.0), showing also a multi-functional behavior working as exochitinase, endochitinase, and N-acetyl-β-D-glucosaminidase [110].

Recently, it was highlighted that *Chromobacterium violaceum* possess a gene encoding a GH18 chitinase [111]. The chitinolytic enzyme (CvChi47) is a thermostable protein with an optimum hydrolytic capacity at 60 °C, retaining approximately 53.7% of its activity at 100 °C for 1 h.

Interestingly, even if there is less information on them, chitinases from Archaea have been exploited for their extreme characteristics. Recently, a chitinase with a dual hydrolytic activity on chitin and cellulose, from the hyperthermophilic anaerobe *Thermococcus kodakarensis* [112], showed thermal stability at 70–80 °C, retaining 40% of its maximum activity at 100 °C.

Two halophilic Archea, *Halobacterium salinarum* and *Haloferax mediterranei*, produce functionally active extracellular chitinases belonging to the GH18 family [113], with a high tolerance at elevated saline concentration.

A chitinase exhibiting high thermostability (95 °C) was found in the hyperthermophilic archaeon *Pyrococcus chitonophagus*, isolated from media containing chitin as the carbon source [114].

#### 5.2.2. Chitin Deacetylase

Chitin deacetylases (CDAs) catalyze the N-deacetylation of insoluble chitin to chitosan; moreover, several deacetylases, named chitooligosaccharide deacetylase (CODs), are active on acetylated chitooligosaccharides (COS), and N-acetyl glucosamine (GlcNAc) [115]. Physico-chemical and biological properties of chitosan and COS are strictly dependent on their degree of polymerisation and fraction of acetylation; thus, the discovery of CDAs with novel specificities, and the definition of new CDA combinations have recently gained considerable attention [116]. Furthermore, chitosan, COS and glucosammine (GlcN), obtained by chitin deacetylase action, are products with enormous biotechnological value; they have a volumetric expected market demand, in 2022, of tens of thousands of tons, finding application in several sectors such as food, medicine, pharma, water treatment, and cosmetic industries [117,118]. CDAs and CODs are metallo-enzymes classified as CE4 enzymes in the CAZy database, with the exceptions of CODs from archaea that belong to the CE14 family [119]. Although CDAs of fungal origin have been the most studied, recently, many other CDAs, and CODs from bacteria and archaea have also been investigated [120].

ArCE4A, a CDA from the marine gram-positive bacterium Arthrobacter sp. AW19M34-1, was studied by Tuveng et al. [109]. ArCE4A has broad substrate specificity, working on COS, chitosan, chitin, and acetyl xylan. Its engineered version with improved catalytic efficiency against crystalline α-chitin showed potential industrial applications for the production of chitosan, and deacetylated COS [121]. BaCDA, a CDA from *Bacillus aryabhattai* B8W22 isolated from sea sediment, was active against chitin, and COS [122]. Its maximum thermostability was detected in the presence of 1 M NaCl at 50 °C and pH 7.0, with 75% residual activity at pH 6.0 and 8.0. Due to its extremophilic nature, BaCDA is considered a promising candidate for industrial exploitation. In addition, terrestrial microorganisms are endowed with chitin deacetylase activity. A CDA, from *Bacillus licheniformis*, revealed biotechnologically relevant properties, being maximally active at 50 °C and pH 7.0, and maintaining a residual activity of 50% at 70 °C and pH 6.0 and 8.0 [123]. Chitin deacetylases, active exclusively on diacetylchitobiose (GluNAc)2 and acetylglucosammine, were studied from Archaea such as *Thermococcus kodakaararensis* KOD1 by Tanaka et al. [124]. The intracellular enzyme (Tk-Dac), a member of the CE14 family, showed biotechnologically relevant features, such as optimal pH, and temperature of 8.5 and 75 °C, respectively.

A deacetylase activity with similar characteristics, but with an optimum pH of 7.5, was found from *Pyrococcus horikoshii* (PhDac) [125]. This property, favorable for the production of N-glucosamine (GlcN), a compound stable under acidic conditions, was improved in the engineered PhDac mutant, showing an optimum pH of 6.0 and a higher specific activity and conversion rate for GlcNAc, compared with the native form [126].

## 6. Conclusions

Over the past two decades, plastid transformation has become a pillar of both molecular and biotechnology research on plastids. Indeed, a very large number of articles have demonstrated the potential of this technology to produce many valuable recombinant proteins, in some cases with extremely high protein accumulation levels, mainly in the tobacco plant. High yields of production, and intrinsic properties of recombinant enzymes, allowed avoidance of time-consuming and expensive purification steps, using plant crude or enriched extracts in biomass conversion [12,30]. Despite many attractive advantages of plastid transformation, in other cases very poor expression levels, phenotypic alteration of transplastomic plants, or low transformation efficiency for crops were observed. To overcome these limitations, many efforts have been carried out to develop protocols for major crops and tools for inducible transgene expression, to identify factors for maximizing expression levels, etc. [14,15]. Recently, several advances have been proposed to simplify and/or shorten the classical chloroplast transformation procedure, but all of them require further optimizations [25,27,28]. Although plastid transformation, mainly in tobacco, has been successfully used to manufacture several cellulolytic enzymes, to date it has not been exploited for the production of auxiliary activities—or other enzymes from bacterial and archaeal microorganisms, with important applications in the valorization of lignocellulosic and/or fishing industry wastes. In fact, the complex structures of such substrates require a multitude of different cell wall degrading activities to be completely depolymerized. In this context, auxiliary activities, such as LPMOs, are gaining particular interest, since they boost cellulose breakdown acting on its recalcitrant crystalline structure by oxidative activity [127]. The possibility of producing cost-effective plant-based enzyme cocktails, that combine complementary activities, will favor the use of enzymes in biomass conversion for biofuel production.

## Figures and Tables

**Table 1:** Strengths and weaknesses of new plastid transformation methods.

Plastid Transformation Method	Strengths	Weaknesses	Reference
Linear DNA fragments	No vector construction required	Sequencing of linear fragments required	[25]
Minichromosome	Stable and efficient amplification of foreign DNA	Need to use transplastomic plants expressing the Rep protein as plant host	[27]
Fusion peptide KH-AtOEP34	Reduction of time-consuming steps	No homoplasmic plants obtained	
	Applicable to recalcitrant crops (rice, kenaf)	Low transformation efficiency	[28]
		Possible integration of exogenous DNA onto nuclear DNA	

## Data Availability

Not applicable.
